# Nature and Trend of Pharmaceutical Payments to Japanese Board-Certified Neurologists Between 2016 and 2019: A Pre-emergence Analysis Amidst the Development of Next-Generation Alzheimer’s Disease Drugs

**DOI:** 10.7759/cureus.53848

**Published:** 2024-02-08

**Authors:** Rajeev Shrestha, Hiroaki Saito, Erika Yamashita, Sunil Shrestha, Tetsuya Tanimoto, Akihiko Ozaki

**Affiliations:** 1 Palliative Care and Chronic Disease, Green Pastures Hospital, Pokhara, NPL; 2 Gastroenterology, Soma Central Hospital, Soma, JPN; 3 Heart Care, Medical Governance Research Institute, Tokyo, JPN; 4 Pharmacy, Monash University Malaysia, Jalan, MYS; 5 Internal Medicine, Navitas Clinic Shinjuku, Tokyo, JPN; 6 Surgery, Graduate School of Public Health, Teikyo University, Tokyo, JPN

**Keywords:** alzheimer’s drugs, neurologist, pharmaceutical payment, japan, industry payment

## Abstract

Objective: There is insufficient data on the financial relationships between Japanese neurologists and pharmaceutical companies prior to the advent of new-generation Alzheimer’s disease drugs. The purpose of this study is to evaluate the magnitude, prevalence, and trend of the financial relationship between Japanese neurologists and pharmaceutical companies between 2016 and 2019.

Methods: A cross-sectional study was undertaken to evaluate the financial relationships between all board-certified neurology specialists and pharmaceutical companies in Japan from 2016 and 2019. Descriptive statistics were applied to measure the magnitude and prevalence of payments among specialists, as well as their trends during the study periods.

Results: In a four-year analysis, 77 pharmaceutical companies disbursed a total of USD 36,869,204 across 50,050 payments to 2,696 neurologists in Japan, revealing a mean payment of USD 10,809 per specialist. Notably, the Gini index of 0.997 indicated a high inequality in payment distribution, with a minority of specialists receiving a substantial proportion of payments. Trends displayed irregularities, but an overall increase in total payments from 2016 to 2019, with a significant contribution from the top 10 pharmaceutical companies accounting for 74.2% of total payments, with Takeda Pharmaceutical and Eisai Company notably increasing payments in 2019. There were notable geographical variations in neurologist and payment distribution across 47 prefectures.

Conclusion: Our analysis of neurologist payments from pharmaceutical companies in Japan showed a substantial financial relationship with overall increases, yearly varied increments, and payment inequality. Caution is warranted as financial ties may intensify with the continued development of next-generation Alzheimer's disease drugs.

## Introduction

Pharmaceutical payment to physicians, often referred to as "pharmaceutical marketing" or "pharmaceutical detailing," is a practice, where pharmaceutical companies provide payments, gifts, or other incentives as a part of their marketing strategies. While this practice can provide opportunities to share pharmaceutical product knowledge and develop innovative treatment approaches for patient care, it also raises concerns about conflicts of interest. These conflicts may lead clinicians to favour their products, which can be problematic and result in negative patient outcomes. This preference can manifest in the form of favoured prescribing, specific treatment guideline recommendations, and biased research [1,2]. Therefore, strict financial support or transaction monitoring between pharmaceutical companies and clinicians is imperative.

Many developed countries have formulated and implemented regulatory policies and guidelines to ensure transparency in the financial transactions between the pharmaceutical industry and physicians [3-5], namely the Physician Payment Sunshine Act and the Open Payments Database in the United States [3,4]. The Japan Pharmaceutical Manufacturers Association (JPMA), representing major pharmaceutical companies in Japan, introduced transparency guidelines in 2013 that require its members to disclose payments made to physicians [5]. Under this regulation, pharmaceutical companies affiliated with the JPMA are required to disclose their payment information on their websites, although the level of detail is less comprehensive than what is disclosed in the United States.

Many previous studies from Japan, Canada, and the United States demonstrated evidence of financial relationships between pharmaceutical companies and physicians of various specialties, such as oncologists, paediatrics, and haematologists, and of various authoritative positions, such as clinical guideline authors, professors, and members of society [1,6-9]. In particular, multiple studies in the United States reported the financial relationship between pharmaceutical companies and neurologists [10,11].

The burden of neurological diseases, especially dementia, is particularly acute in developed countries experiencing population aging, and Japan is no exception. As of September 15, 2022, individuals aged 65 years or older constituted 29.1% of the Japanese population, being the highest elderly population in the world [12]. Indeed, Ikeda et al. estimated an average of JPY 1,073 billion annual healthcare cost for Alzheimer’s disease dementia, one of the common neurological conditions in Japan [13]. Currently, we are witnessing the emergence of next-generation Alzheimer's disease drugs, such as lecanemab, developed by the Japanese pharmaceutical company Eisai, which costs nearly three million yen per patient per year [14]. It is anticipated that associated payments will rise in the future, affecting Japan and beyond. In light of this situation, it is important to understand the trends in payments before the widespread introduction of these new therapeutic agents and prepare for the future upsurge. This study explored the pharmaceutical payment magnitudes and trends in Japan from 2016 to 2019.

## Materials and methods

Study design

This cross-sectional retrospective analysis evaluates the financial relationships between all board-certified neurology specialists in Japan and pharmaceutical companies. The study population comprises all neurology specialists certified by the Japanese Society of Neurology (JSN). JSN is the sole and largest professional medical society for neurology in Japan, which trains and certifies neurologists with extensive skills and knowledge.

Data collection

We extracted specialists’ names and affiliations from the official JSN webpage on December 31, 2021. We obtained information on the prefectures of affiliated facilities associated with the data provided in this study. Additionally, we collected details of payments (speaking/lecturing, writing, consulting, etc.) made by all JPMA-member pharmaceutical companies to healthcare specialists between 2016 and 2019. This data collection was facilitated by the JPMA transparency guidance, a voluntary initiative promoting the disclosure of payments to healthcare professionals and organizations. As of December 2021, these collected data represented the latest publicly available dataset in Japan. Information on payments for speaking, writing, and consulting was available at the individual physician level. Notably, smaller and more common payment categories (meals & beverages, travel/accommodation, trial enrolment reimbursements) were not individually disclosed by companies, as previously mentioned.

Data analysis

Payment values and counts were analysed descriptively for both specialists and pharmaceutical companies. Subsequently, the Gini index, a measure of income inequality ranging from 0 to 1, was employed to assess payment concentration among specialists. Higher Gini values indicated a greater disparity in payment distribution. Furthermore, this study examined trends in physician payments from pharmaceutical companies in Japan. Payment data were analysed for companies participating in the JPMA over a four-year period. The relative percentage of the average annual increase in payments per specialist and the number of specialists receiving payments were also calculated. Payment trends were assessed for years of payments, number of recipient physicians, and individual payment values. To explore regional variations in physician-industry relationships, we aggregated payments and specialist numbers by prefecture of the physician's affiliated facility. We then calculated and compared payment amounts and neurologist numbers per million people in each prefecture. Population data were based on the October 1, 2019, Basic Resident Register. The payment values were expressed in USD dollars using the 2019 average monthly exchange rate, JPY 109.0 per USD 1. Analyses were conducted with Microsoft Excel 16.0 (Microsoft® Corp., Redmond, WA) and Python 3.9.10.

Ethical approval

This study received ethical approval from the Medical Governance Research Institute (approval number: MG2018-04-20200605; date: June 5, 2020). Due to its reliance on publicly available data from pharmaceutical companies and the society webpage, informed consent was waived by the Ethics Committee.

## Results

Overview of payments

**Figure 1:**
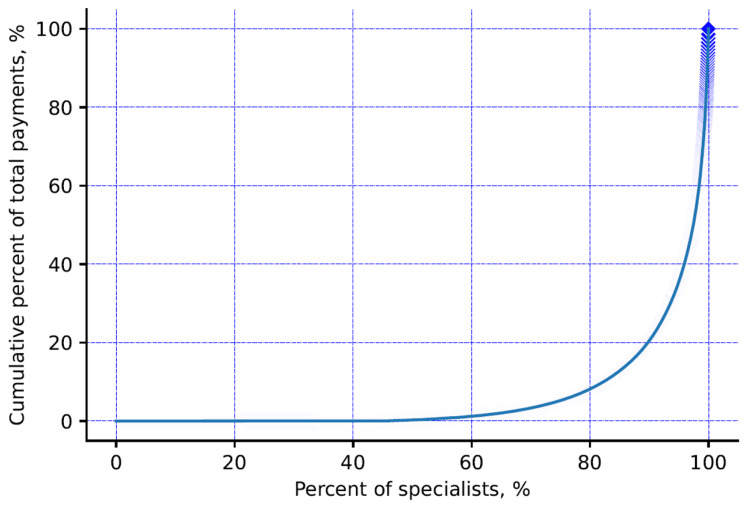
Pharmaceutical payment concentration among neurologists in Japan

Out of a total of 6,107 registered neurologists under the Japanese Society of Neurology, only 3,411 (55.9%) neurologists received a total of USD 36,869,204 (JPY 4,018,743,154) from 77 pharmaceutical companies with 50,050 payment counts. Among the 93 companies, 15 companies did not pay money for the physician included in the study. The mean (standard deviation, SD) and median (interquartile range, IQR) four-year combined payment values per specialist were USD 10,809 (SD: USD 29,791) and USD 2,214 (IQR: USD 715-7,690), respectively. At maximum, one specialist received USD 502,645 personal payments over the four years. The mean and median number of counts over the four years were 15 (SD: 29) and five (IQR: 2-14) counts per specialist. The neurologists received payments from an average of 8.9 (SD: 4.3) and a median of 4.0 (IQR: 2-11) pharmaceutical companies. The maximum payment counts and pharmaceutical companies per specialist over the four years were 355 payments and 119 companies. The Gini index was reported at 0.997 for the four-year combined total payments per specialist, indicating high inequality in payment distribution among neurologists. A very low proportion of specialists are receiving a high proportion of payment (Figure 1). The most common payment category was speaking, which occupied 84.8% (USD 31,272,630) of total payments (Table 1).

**Table 1:** Summary of personal payments from pharmaceutical companies to neurologists from 2016 to 2019

Variables	Content	Value
Total payments, USD	Payment values, USD	36,869,204
	Counts, n	50,050
	Companies, n	77
Average per specialist ± SD	Payment values, USD	10,809±29,791
	Counts, n	15±29
	Companies, n	8.9
Median (IQR)	Payment values, USD	2,214 (715-7,690)
	Counts, n	5 (2-14)
	Companies, n	4 (2-11)
Range	Payment values, USD	31-502,645
	Counts, n	1-355
	Companies, n	1-119
Physicians with specific payments, n (%)	Any payments	3,411 (55.9)
	Payments>USD 500	2,950 (48.3)
	Payments>USD 1000	2,334 (38.2)
	Payments>USD 5000	1,137 (18.6)
	Payments>USD 10000	714 (11.7)
	Payments>USD 50000	155 (2.5)
	Payments>USD 100000	70 (1.1)
Gini index		0.997
Category of payments, USD (%)	Speaking	31,272,630 (84.8)
	Consulting	4,154,762 (11.3)
	Writing	1,430,748 (3.9)
	Other	10,860 (0.0)
Note: SD: Standard Deviation; n: number, IQR; interquartile range		

Trends of payments

Table 2 presents the trend of pharmaceutical payments to neurologists from 2016 to 2019. The median payments per specialist ranged from USD 1,055 (IQR: USD 511-3,319) in 2016 and USD 1,331 (IQR: USD 520-3,772) in 2019. The annual change rate was 2.6% for all pharmaceutical companies for four years; however, it was only 1% while specifically reviewing the complete four-year pharmaceutical payment data. The Gini index is uniform in all years in the four-year complete database group, while the index increased in 2019, viewing the overall payment scale. The yearly payment increase was the highest in the specialist group receiving more than USD 100,000.

**Table 2:** Trend of personal payments from pharmaceutical companies to neurologists from 2016 to 2019

Variables	Content	2016	2017	2018	2019	Average yearly change, %
All pharmaceutical companies (n=77)						
Total payments	USD (JPY)	8,633,140 (941,012,221)	9,213,717 (1,004,295,168)	9,220,946 (1,005,083,103)	9,801,197 (1,068,330,426)	
Average payments ± SD	USD	3,962±9,439	4,102±9,668	4,107±9,140	4,276±9,262	2.6
Median payments	USD	1,055 (511-3,319)	1,226 (511-3,270)	1,328 (511-3,576)	1,331 (520-3,772)	
Payment range	USD	58-110,581	0-134,906	31-138,830	68-144,195	
Gini index		0.997	0.997	0.997	0.998	
Physicians with specific payments, n (%)	Any payments	2,179 (36.0)	2,242 (37.0)	2,245 (37.0)	2,292 (38.0)	1.7
	Payments>USD 500	1,751 (29.0)	1,863 (31.0)	1,885 (31.0)	1,936 (32.0)	3.4
	Payments>USD 1,000	1,196 (20.0)	1,285 (21.0)	1,344 (22.0)	1,378 (23.0)	4.9
	Payments>USD 5,000	376 (6.2)	410 (6.7)	414 (6.8)	446 (7.3)	5.9
	Payments>USD 10,000	188 (3.1)	211 (3.5)	207 (3.4)	221 (3.6)	5.7
	Payments>USD 50,000	23 (0.38)	19 (0.31)	24 (0.39)	19 (0.31)	-4.0
	Payments>USD 100,000	3 (0.05)	4（0.07）	1 (0.02)	2 (0.03)	19.4
Pharmaceutical companies with 4-year payment data (n=46)						
Total payments	USD (JPY)	8,534,024 (930,208,577)	9,138,961 (996,146,764)	8,737,552 (952,393,158)	9,028,687 (984,126,840)	
Average payments ± SD	USD	3,955±9,416	4,122±9,659	3,952±8,895	4,061±8,865	1.0
Median payments	USD	1,055 (511-3,317)	1,226 (511-3,275)	1,276 (511-3,401)	1,328 (520-3,631)	
Payment range	USD	58-110,581	0-132,862	31-134,241	68-141,086	
Gini index		0.997	0.997	0.997	0.997	
Physicians with specific payments, n (%)	Any payments	2,158 (35.0)	2,213 (36.0)	2,211 (36.0)	2,223 (36.0)	1.0
	Payments>USD 500	1,730 (28.0)	1,845 (30.0)	1,858 (30.0)	1,881 (31.0)	2.9
	Payments>USD 1,000	1,184 (19.0)	1,279 (21.0)	1,323 (22.0)	1,339 (22.0)	4.2
	Payments>USD 5,000	373 (6.1)	408 (6.7)	390 (6.4)	410 (6.7)	3.4
	Payments>USD 10,000	188 (3.1)	211 (3.5)	189 (3.1)	196 (3.2)	1.8
	Payments>USD 50,000	22 (0.4)	19 (0.3)	21 (0.3)	16 (0.3)	-9.0
	Payments>USD 100,000	3 (0.0)	4 (0.1)	1 (0.0)	2 (0.0)	19.4

Payments by company

**Figure 2:**
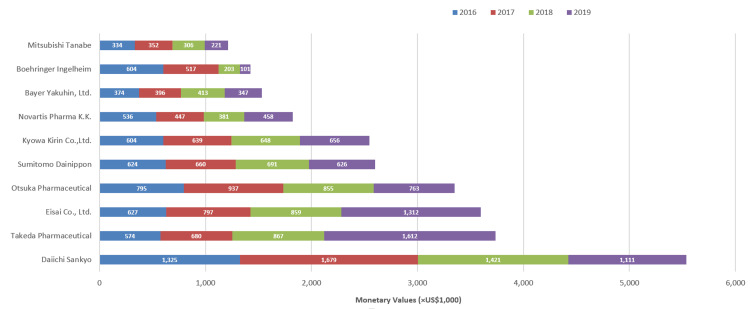
Top 10 largest-paying pharmaceutical companies

The payment types by the top 10 paying companies are shown in Figure 2. Among 77 paying pharmaceutical companies, payments from the top 10 companies accounted for 74.2 % of total payments, with USD 27,355,989 between 2016 and 2019. Daiichi Sankyo was the top company providing the largest payment in all years, except in 2019. However, in recent years, two companies (Takeda Pharmaceutical and Eisai Company) have drastically increased their payments in 2019 compared to previous years, securing the highest-paying company in 2019.

Distribution of payment and specialists by prefecture

**Figure 3:**
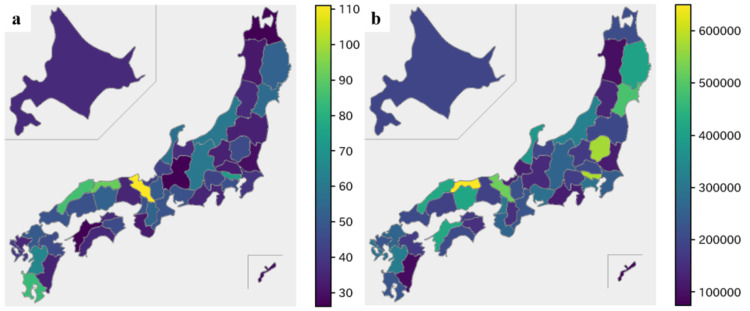
Schematic distribution of neurologists and payment based on prefecture – (a) number of neurologists per million people and (b) payment amounts per million people in USD

There were notable geographical differences in the distribution of neurologists and payment in Japan (Figure 3). Kyoto (111.11 per million people) and Tottori (91.73 per million people) were the top two prefectures having the highest proportion of neurology specialists’ distribution, while Tottori (USD 649,999) and Tokyo (USD 579,634) had the highest payment distribution. Similarly, the lowest payment distribution was in Okinawa (USD 74,461) and Miyazaki (USD 94,236), while the lowest neurologist distribution was different, in Ehime (26.14 per million people) and Gifu (26.17 per million people). The details of payment and specialist distribution among prefectures are given in the attached supplementary file (see Appendix, Table 3).

## Discussion

This study is the first to record the pharmaceutical payment trend to neurologists in Japan. This study found that a total of 36,869,204 US dollars was received by Japanese neurologists from 2016 to 2019, with 10,809 US dollars as an average payment per specialist from 8.9 companies. Many previously conducted pharmaceutical payment studies have also shown a huge payment trend in Japan from 2016 to 2019: $53,547,391 to respiratory specialists [6], $33,223,806 to dermatologists [15], $908,900 to paediatric haematologists/oncologists [16], and $36,291,434 to haematologists [17]. Furthermore, Ahlawat et al. [11] reported that $6,210,414 was given to American neurologists for non-research purposes, with an average of $891 in 2015, and Nalleballe et al. [10] reported an average of $627 in 2013 and $3,396 in 2018 to vascular neurologists of the United States. Although a clear picture of payment to neurologists cannot be drawn globally due to the limited studies on pharmaceutical payments to neurologists, the overall findings reported to date showed huge investment in pharmaceutical payments to neurologists, even before the launch of the new Alzheimer’s disease agents. Similarly, there is a lack of detailed documentation on payment; for example, American studies reported payments for research or non-research purposes.

Furthermore, the overall payment of neurologists increased from 2016 to 2019, with an average yearly increment of 2.6%, which is comparatively very low compared to yearly payment increase among other fields of specialties in Japan, such as respiratory specialists (7.6%) [18], head and neck surgery specialists (12.4%) [19], dermatologists (14.1%) [15], and gastroenterologists (2.4%) [20], but similar to haematologists (1.1%) [17]. Similarly, two studies on payment to vascular neurologists and neurology subspecialties in America showed an increasing payment trend. Non-research annual payments to vascular neurologists in the United States increased from $99,749 in 2013 to $1,032,302 in 2018, while the receiving neurologist proportion increased only by 1% [10]. The overall industry payment to neurology subspecialties increased by 16% from 2014 to 2018 in the United States [21]. The higher payments observed in the US compared to Japan may be attributed to the inclusion of payments from medical device companies to neurologists in the US data, whereas our study only accounted for payments from pharmaceutical companies [22]. Actually, there is a lack of data on payment by the medical device industry in Japan. Penetration of newer medicines in the neurology market was assumed to be the influencing factor for increased payment in the United States [21] and in previous studies of Japan [23], which could also be a potential cause in our context.

One of the prominent reasons for industry payment to prescribers is to increase the market consumption of their product by prescribing by prescribers. Therefore, many studies illustrated that the sponsorship for education interactions (for example, educational training and food and beverage) and consultation fees increase prescribing [24,25]. In our study, most of the payment was given for speaking ($31,272,630,84.8%), followed by consulting (4,154,762,11.3%), which is consistent with previous studies of Japan [6,17,19,23]. Currently, very few countries are only presenting pharmaceutical payments publicly. Limited database studies of US neurologists also showed higher payment distribution for consultation fees and lower for activities such as food and beverages, around 10-11.5% of the total payment amount [10,26]. Ahlawat et al. [26] specified that the most common payment was for food and beverages (86.5%) but covered a total of 11.5% of total payments. Therefore, further detailed exploration of activities and payment purposes needed to be explored to understand the potential impact and implication of increasing payment trends in Japan.

Similar to the previous studies of Japan, this study also showed the uneven distribution of payment to specialists, irrespective of the geography and population of the prefecture [6,17]. Half of the specialists received less than $1,000, while only 1.1% received more than $100,000 payments yearly. This is very similar to the payment studies among head and neck surgeons [19], dermatologists [15], gastroenterologists [20], and respiratory physicians [6], but higher compared with haematologists [17] in Japan. For example, Nalleballe et al. reported a higher distribution of payment to neuroimmunology/MS specialties in the United States [21]. The most significant reason for not being able to use detailed specialties of neurologists in our study is the failure to evaluate positions at their institutions, positions in academic societies, positions in guidelines and academic activities, etc. Past studies have shown that monetary donations are concentrated on physicians who hold important and influential positions in guidelines and academic societies in Japan [20,27,28], and it is possible that the same is true in neurology. Furthermore, the diverse payment distribution could be the result of high payment by the selected pharmaceutical companies producing specific medicines targeting specific specialists. Out of 77 paying pharmaceutical companies, only 10 cover 74.2% of total payments ($27,355,989), where Daiichi Sankyo was the top company providing the highest payment amount in all years, except in 2019. This is the same company that provided the highest research payment to neurologists in America in 2015 [11]. Meanwhile, two companies, Takeda Pharmaceutical and Eisai Company, have drastically increased their payments in 2019 compared to Daiichi, which can change the payment distribution pattern in the coming years with the possibility of securing the highest payment position. This can be expected because they have already started launching a few medicines targeting neurologists, such as lemborexant and perampanel by Eisai company and a combination of vonoprazan aspirin by Takeda [29,30].

Limitation

This study retains some limitations with areas to explore in the future. First, the study utilized the publicly available database for four years; therefore, there could be the possibility of underreporting or not all the pharmaceutical companies disclosing their payment details. Secondly, this study has not explored the details of payment distribution patterns in neurologists and the causes for that. The detailed information on payment purposes and payments for specific groups or categories of medicines would help explore and have a comprehensive understanding of payment trends uncovered in this study due to limited access to data. Furthermore, the monetary inflation rate during the four-year period (2016-2019) in Japan was not evaluated and adjusted while calculating the payment values.

## Conclusions

Our analysis of personal payments from pharmaceutical companies to neurologists in Japan from 2016 to 2019 reveals a total disbursement of USD 36,869,204 across 50,050 payment instances to 2,696 neurologists. Despite an overall increase in payments, yearly increments are highly unequal. Only a very low proportion of specialists are receiving a high amount of payment. Geographical analysis highlighted differences in neurologist and payment distribution across prefectures. Our findings provide insights into the landscape of personal payments in neurology, emphasizing trends, disparities, and the influential role of top-paying pharmaceutical companies in this financial dynamic. Considering the anticipated increase in payments from pharmaceutical companies due to the development of novel Alzheimer’s disease treatments, it is essential to understand the current financial landscape within the neurology field and prepare for this expected upsurge.

## Appendices

**Table 3:** Details of the distribution of the number of specialists per million people and payment amount per million people in all provinces of Japan

S.N.	Prefecture	Number of specialists per million people	Payment amount per million people
1	Aichi Prefecture	47.66949153	289279.1654
2	Akita	32.09109731	97107.18559
3	Aomori Prefecture	26.4847512	209676.2189
4	Chiba Prefecture	39.46317303	244916.2835
5	Ehime Prefecture	26.13890963	421497.6328
6	Fukui Prefecture	42.96875	129207.0671
7	Fukuoka Prefecture	50.15673981	262631.5949
8	Fukushima Prefecture	34.12784399	199886.4791
9	Gifu Prefecture	26.17010569	146132.268
10	Gunma Prefecture	33.98558187	172523.8239
11	Hiroshima	47.07560628	186106.0477
12	Hokkaido	36.38095238	195209.5955
13	Hyogo Prefecture	35.49213319	186255.2963
14	Ibaraki Prefecture	29.72027972	117891.602
15	Ishikawa Prefecture	57.11775044	378821.3508
16	Iwate Prefecture	52.97473513	406652.2883
17	Kagawa Prefecture	36.61087866	172930.7992
18	Kagoshima Prefecture	83.6454432	226175.3141
19	Kanagawa Prefecture	47.18417047	296697.4492
20	Kochi Prefecture	35.81661891	206781.6566
21	Kumamoto Prefecture	64.64530892	316708.957
22	Kyoto	111.1111111	524038.4057
23	Mie Prefecture	48.28747894	229562.6722
24	Miyagi Prefecture	55.94102342	484496.5785
25	Miyazaki Prefecture	34.48275862	94235.93286
26	Nagano Prefecture	60.02928258	260829.2208
27	Nagasaki Prefecture	40.69329314	289935.1438
28	Nara Prefecture	53.38345865	153534.7313
29	Niigata Prefecture	60.27890238	326590.8744
30	Oita Prefecture	45.81497797	174123.9623
31	Okayama Prefecture	52.38095238	413013.6498
32	Okinawa Prefecture	27.52924983	74461.12125
33	Osaka Prefecture	48.0190714	304155.3436
34	Saga Prefecture	35.58282209	225569.0325
35	Saitama	32.5170068	206139.8465
36	Shiga Prefecture	50.21216407	220069.813
37	Shimane Prefecture	86.05341246	421448.5204
38	Shizuoka Prefecture	34.8518112	123658.9291
39	Tochigi Prefecture	46.01861427	572870.3832
40	Tokushima	45.32967033	157042.4942
41	Tokyo	73.05509662	579634.3133
42	Tottori Prefecture	91.72661871	649998.68
43	Toyama Prefecture	37.35632184	197223.1977
44	Wakayama Prefecture	32.43243243	262027.0965
45	Yamagata Prefecture	36.17810761	135775.0847
46	Yamaguchi Prefecture	48.60088365	203510.62
47	Yamanashi Prefecture	43.15659679	181234.1542
